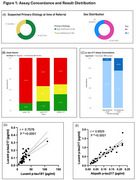# Clinical Utility of Plasma Biomarkers of Alzheimer's Disease (CliPAD)

**DOI:** 10.1002/alz70861_108880

**Published:** 2025-12-23

**Authors:** Juli Cehula, Firoza Z Lussier, Marina Scop Madeiros, Pamela C.L. Ferreira, Tharick A Pascoal

**Affiliations:** ^1^ University of Pittsburgh, Pittsburgh, PA USA

## Abstract

**Background:**

Clinical adoption of blood‐based biomarkers for Alzheimer’s disease (AD) requires real‐world validation. The CliPAD study aims to assess the clinical utility of plasma phosphorylated‐tau (*p* ‐tau) biomarkers by evaluating their diagnostic performance, concordance with amyloid‐β (Aβ) PET imaging, and impact on treatment decisions in routine care.

**Method:**

CliPAD is an observational study enrolling up to 200 participants (ages 40–90) referred through UPMC clinics. Eligible individuals have objective cognitive impairment. The study visit includes blood sampling and rapid cognitive testing (MoCA), with questionnaires (NPI‐Q, AD8, FAS) completed by a study partner. Clinical plasma *p* ‐tau assays (Lucent *p* ‐tau181, Lucent *p* ‐tau217, ALZpath *p* ‐tau217) are performed in CLIA‐certified laboratories and results are returned to the referring clinician. Post‐assay medical care is monitored to evaluate impacts on clinical management. Optional follow‐ups occur at 12, 24, and 36 months.

**Result:**

Seventy‐eight referrals were received from 14 clinicians within the UPMC Neurology and Geriatric Medicine departments. N=52 participants (mean age: 70.8 ± 11.6 years; MoCA score: 18.3 ± 6.6) are currently enrolled. At referral, primary etiology is recorded as high or low likelihood of AD per clinical opinion of the referring provider (Figure 1A). Figure 1B shows the frequency of positive, intermediate, and negative across *p* ‐tau assays. The *p* ‐tau217 results show a stronger correlation (r=0.9525) compared to *p* ‐tau217 (Lucent) and *p* ‐tau181 (r=0.7576) (Figures 1D, E). Discordance between *p* ‐tau217 assays was greater in individuals the low‐likelihood AD group (Figure 1C).

To date, N=11 participants have undergone Aβ PET imaging post assay results. Currently, N = 7 are receiving anti‐amyloid therapy (*N* =6 Leqembi, N=1 Kisunla), all with concordant positive *p* ‐tau assays. Additionally, N=10 have been referred for Aβ PET imaging; N=6 demonstrated concordant assay results, while N=4 exhibited discordances. Pending Aβ PET imaging results, ant‐amyloid treatment may be recommended.

**Conclusion:**

The greater discordance between *p* ‐tau217 assays in the low‐likelihood AD group (Figure 1C) highlights the complexity of application of these biomarkers in clinical practice. These findings emphasize the need for further research in clinical settings to refine their use for accurate diagnosis and informed treatment decisions.